# Synergistic Effects of Nitrogen and Zinc Foliar Application on Yield and Nutrient Accumulation in Rice at Various Growth Stages

**DOI:** 10.3390/plants13233274

**Published:** 2024-11-22

**Authors:** Patcharin Tuiwong, Hui-Kyong Cho, Hatem Rouached, Chanakan Prom-U-Thai

**Affiliations:** 1 Agronomy Division, Department of Plant and Soil Sciences, Faculty of Agriculture, Chiang Mai University, Chiang Mai 50200, Thailand; tuiwongp8@gmail.com; 2 Department of Plant, Soil, and Microbial Sciences, Michigan State University, East Lansing, MI 48824, USA; chohuiky@msu.edu; 3 Plant Resilience Institute, Michigan State University, East Lansing, MI 48824, USA; 4 Lanna Rice Research Center, Chiang Mai University, Chiang Mai 50100, Thailand

**Keywords:** foliar nutrient spraying, rice yield, nitrogen (N), zinc (Zn), nutrient uptake, gene expression, Zn transporters, OsZIP genes, nutritional quality

## Abstract

The rising interest in foliar nutrient spraying as a strategy to boost crop yields has led to investigations of how such application influences nutrient uptake and accumulation, especially in edible plant parts. Despite its importance, the effects of single versus simultaneous nutrient application on plant absorption, transport, and accumulation have been underexplored. This study addresses this knowledge gap by examining the physiological and molecular responses of rice to foliar application of nitrogen (N) and zinc (Zn) individually and in combination at different growth stages. We assessed how the treatments affect rice grain yield and nutrient accumulation in relation to the expression of Zn transport-related genes. Foliar application of N+Zn+ at the tillering stage resulted in a 62.01% increase in grain yield compared to the control. Additionally, Zn concentrations in brown rice were increased by 26.04% and 34.20% when N0Zn+ and N+Zn+ treatments, respectively, were applied at panicle initiation. Gene expression analysis revealed that the timing and nutrient combination significantly influenced rice productivity and grain Zn concentration. At the tillering stage, the N+Zn+ treatment elevated the expression of Zn transporters such as *OsZIP3*, *OsZIP4*, and *OsZIP9* in leaves, thereby enhancing grain yield. At panicle initiation, the nutrient treatments influenced a broad range of genes, including *OsZIP4*, *OsZIP9*, *OsHAM2*, *OsDUR3*, *OsAAP1*, *OsGS1;1*, and *OsFd-GOGAT*, affecting grain Zn and N accumulation. These insights are crucial for developing targeted nutrient management strategies to optimize rice yield and grain nutritional quality for the benefit of consumers.

## 1. Introduction

Zinc (Zn) is an essential micronutrient that is critical for numerous physiological and biochemical processes in plants, including enzyme activation, protein synthesis, and growth regulation [1]. Zn deficiency is a concerning global issue, particularly in regions with cereal-based diets, where Zn intake is often inadequate [2]. Agronomic biofortification, a process employed to increase the nutrient content of crops, can combat Zn deficiency and enhance crop nutritional quality [3]. Traditional soil application of Zn is commonly used, although foliar application has emerged as a promising alternative. However, the optimal timing, the appropriate developmental stages for application, and the interactions with other nutrients remain inadequately understood.

Recent advances have revealed the intricate processes involved in Zn uptake and homeostasis, encompassing soil uptake, radial transport through root tissues, and long-distance translocation to shoots and reproductive organs [4]. Zinc homeostasis is orchestrated by diverse transporter proteins, including ZRT/IRT-like proteins (ZIPs), heavy metal ATPases (HMAs), and metal tolerance proteins (MTPs) [5]. Notable transporters such as *OsZIP4* and *OsHMA2* are crucial for Zn uptake, translocation to the panicle, and grain accumulation, underscoring their significance in optimizing Zn levels [6,7,8].

Nitrogen (N) is a crucial macronutrient essential for plant physiology and nutrient interactions, playing a central role in processes such as protein synthesis, enzyme production, and overall plant growth. Key genes involved in N metabolism include *OsDUR3*, which is critical for ureide metabolism; *OsAAP1*, which facilitates amino acid transport; *OsGS1;1*, responsible for synthesizing glutamine; and *OsFd-GOGAT*, which is essential for converting 2-oxoglutarate into glutamate [9,10,11]. These genes are pivotal for N assimilation and utilization, and their functions and interactions with other nutrients, such as Zn, warrant further investigation. Understanding how plants coordinate signals from both N and Zn to regulate their nutrient accumulation, growth, and nutritional quality, particularly in response to foliar application, remains an area of limited exploration.

Foliar application is a precise method of enhancing nutrient availability and uptake, often providing superior results compared to traditional soil application [1]. Despite its potential, the interaction between foliar-applied Zn and N is an emerging but relatively unexplored field. While N significantly influences various aspects of plant metabolism, including the synthesis of proteins and enzymes involved in Zn transport, the specific impacts of foliar-applied N on Zn homeostasis are poorly understood [12]. Further research is needed to clarify these interactions and improve nutrient management strategies for optimizing plant health and productivity.

This study aims to address these knowledge gaps by investigating the interaction effects of foliar application of Zn and N on rice during key growth stages at tillering and panicle initiation. The research will assess the impact of these treatments on grain yield, nutrient concentrations in plant tissues, and the expression of genes associated with Zn and N homeostasis. By elucidating the interactions between foliar-applied Zn and N, this study seeks to optimize nutrient management strategies, potentially influencing agricultural practices and policies designed to enhance rice yield and nutritional quality. The results will contribute to a broader understanding of nutrient management in the context of global food security and sustainable agricultural practices.

## 2. Results

### 2.1. Enhanced Rice Yield with Combined Foliar N and Zn Application

**Figure 1:**
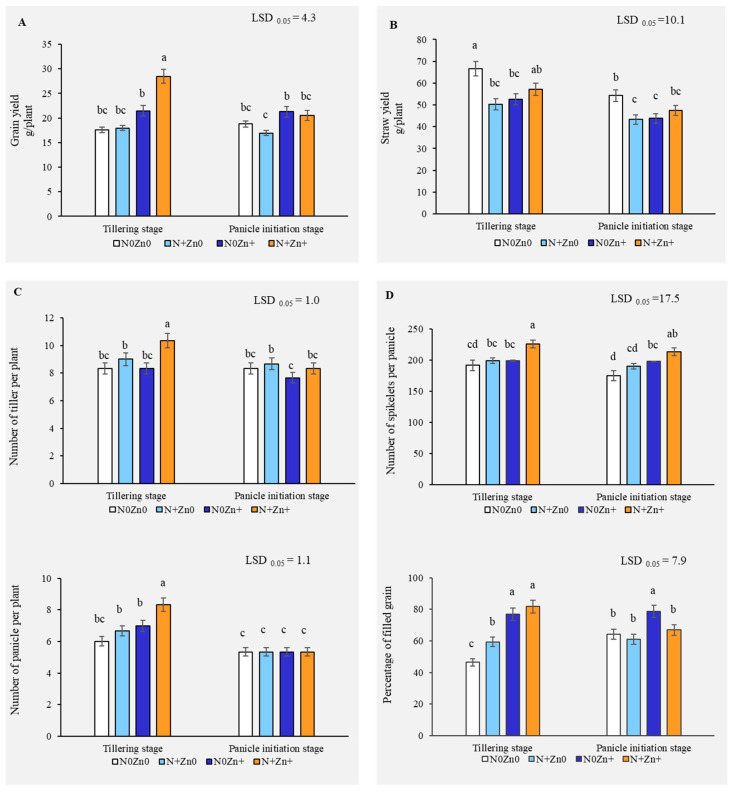
Grain yield (**A**), straw dry weight (**B**), tillers and panicles number per plant (**C**), number of spikelets per panicle, and percentage of filled grain per plant (**D**) of an indica rice variety grown under four foliar application treatments of N and Zn at different growth stages. Each result represents the average from three independent replications. Different letters above the bars indicate significant difference effect of foliar applications of N and Zn by LSD 0.05 at *p* < 0.01. Bars are the standard error of the mean (SE) in each treatment (n = 3).

We investigated the impact of combined foliar application of N and Zn on rice productivity, focusing on grain yield, straw biomass, and yield components. The application of N and Zn together at the tillering stage resulted in a significant 62.01% increase in grain yield compared to the control, an increase that was not observed with the individual applications of N or Zn (Figure 1A). This indicates the necessity of the synergistic interaction effects of both nutrients to maximize yield. Concurrently, straw dry weight was reduced by 24.55% and 21.12% with the individual applications of N and Zn, respectively, at the tillering stage, and the reduction persisted through to the panicle initiation stage (Figure 1B). Moreover, the number of tillers and panicles per plant increased significantly by 24.00% and 38.89%, respectively, with the combined N+Zn+ treatment, highlighting its effectiveness in promoting vegetative growth and panicle development (Figure 1C). The number of spikelets per panicle was also enhanced by 17.82% at the tillering stage by N+Zn+, and further increases were observed at the panicle initiation stage, with N0Zn+ and N+Zn+ treatments improving spikelets by 12.81% and 21.75%, respectively (Figure 1D). Additionally, the percentage of filled grains showed substantial improvements, with the combined N+Zn+ treatment leading to a 76.21% increase compared to the control at the tillering stage and 22.35% at the panicle initiation stage. These findings underscore the critical role of combined N and Zn application in enhancing rice yield, growth, and grain quality while also influencing biomass allocation.

### 2.2. Foliar N+Zn+ Treatments Significantly Improved Zn and N Accumulation in Rice Grains

**Figure 2:**
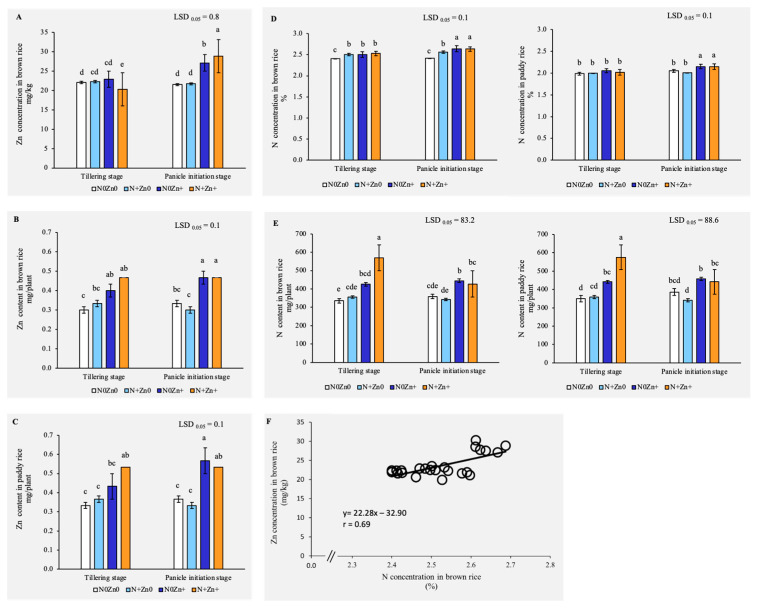
Zinc concentration (**A**) and content (**B**) in brown rice, Zn content in paddy rice (**C**), N concentration in brown rice and paddy rice (**D**), and N content in brown rice and paddy rice (**E**) of an indica rice variety grown under four foliar application levels of N and Zn at different growth stages. The results represent the averages from three independent replications. Different letters above the bars indicate significant differences in the effect of foliar application of N and Zn by LSD 0.05 at *p* < 0.01. Bars are standard errors of mean (SE) in each treatment (n = 3). (**F**) The correlation (r) between Zn concentration and N concentration in brown rice (n = 24) (*p* < 0.01).

To evaluate the effects of foliar treatments on nutrient accumulation in rice, we assessed Zn and N concentrations and contents in both brown and paddy rice. Our results indicated that foliar application of Zn, particularly in combination with N (N+Zn+), significantly enhanced Zn and N levels in rice grains (Figure 2A). At the panicle initiation stage, the Zn concentration in brown rice was increased by 26.04% and 34.20% by N0Zn+ and N+Zn+ treatments, respectively, compared to the control, while the Zn content in brown rice showed significant increases of 33.33% and 55.57% with the same treatments at the tillering stage (Figure 2A,B). In paddy rice, the Zn content was elevated by 54.54% with N0Zn+ and by 45.43% with N+Zn+ at the panicle initiation stage, with an even greater increase of 60.01% observed with N+Zn+ at the tillering stage (Figure 2C). Similarly, the N concentration was the highest with the N0Zn+ and N+Zn+ treatments, showing increases of 9.38% and 9.10% in brown rice and 4.84% and 4.87% in paddy rice, respectively, at the panicle initiation stage (Figure 2D). The N content in brown rice was increased by 26.94% and 69.68% with N0Zn+ and N+Zn+ treatments at the tillering stage, respectively, and by 26.31% and 64.54% in paddy rice (Figure 2E). Additionally, there was a positive correlation between Zn and N concentrations in brown rice (r = 0.69, *p* < 0.05), suggesting a synergistic interactive effect of these nutrients on grain accumulation (Figure 2F). These findings highlighted the effectiveness of Zn and N foliar treatments in improving nutrient uptake and grain quality, emphasizing the importance of balanced nutrient management in rice cultivation.

### 2.3. Combined N and Zn Treatment Enhanced Gene Expression for Nutrient Uptake and Metabolism in Rice

**Figure 3:**
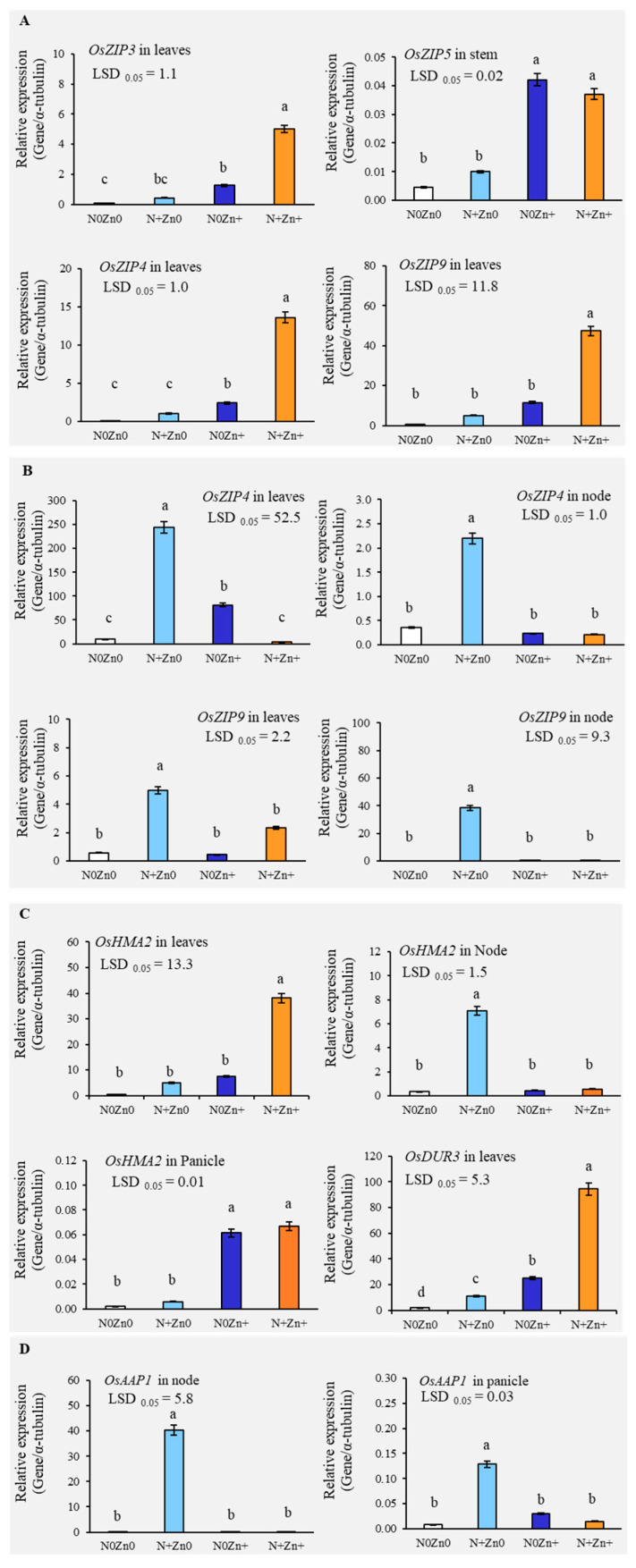
(**A**) The relative gene expression levels of *OsZIP3*, *OsZIP4*, and *OsZIP9* in leaves and *OsZIP5* in stems of an indica rice variety grown under four foliar application treatments of N and Zn at the tillering stage. (**B**) The relative gene expression level of *OsZIP4* and *OsZIP9* in leaves and nodes grown under foliar application at the panicle initiation stage. (**C**) The relative gene expression levels of *OsHMA2* and *OsDUR3* in leaves grown under foliar application at the tillering stage and *OsHMA2* in nodes and panicles grown under foliar application at the panicle initiation stage. (**D**) The relative gene expression level of *OsAAP1* in nodes and panicles grown under foliar application at the panicle initiation stage. Each result represents the average of three independent replications. Different letters above the bars indicate significant differences by LSD 0.05 at *p* < 0.01. Bars are standard errors of means (SE) in each treatment (n = 3).

To elucidate the molecular mechanisms underlying the observed enhancements in plant growth, yield, and nutrient accumulation, we performed gene expression analysis focusing on key genes involved in Zn and N transport, metabolism, and uptake. The objective was to understand how foliar treatments influence physiological and biochemical pathways in rice plants at the molecular level. Our results revealed that the combined N+Zn+ application significantly upregulated the expression of several Zn transporter genes, including *OsZIP3*, *OsZIP4*, *OsZIP5*, and *OsZIP9*, particularly at the tillering stage (Figure 3A). Notably, *OsZIP3* and *OsZIP4* exhibited markedly higher expression levels under treatment with N+Zn+ compared to the control, indicating enhanced Zn uptake and transport. At the panicle initiation stage, *OsZIP4* and *OsZIP9* showed elevated expression in leaves and nodes, with the N+Zn0 treatment yielding the highest levels (Figure 3B).

Regarding Zn and N metabolism genes, *OsHMA2*, which facilitates Zn delivery to developing tissues, displayed a significant 116.37-fold increase with N+Zn+ at the tillering stage (Figure 3C). The expression level of this gene was also notably elevated in the nodes by N+Zn0 at the panicle initiation stage and in the panicle stage by both N0Zn+ and N+Zn+ treatments (Figure 3C). For N metabolism, the expression of *OsDUR3*, a crucial gene in N translocation, was increased up to 45.06-fold in leaves by the N+Zn+ treatment at the tillering stage (Figure 3C). Additionally, *OsAAP1*, responsible for amino acid uptake, showed dramatic increases in expression, particularly with N+Zn0 at the panicle initiation stage (Figure 3D).

**Figure 4:**
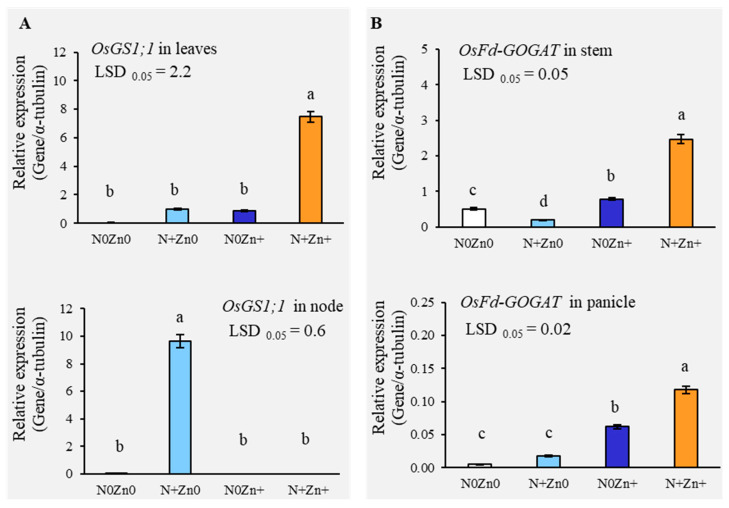
(**A**) The relative gene expression level of *OsGS1;1* in leaves of an indica rice variety grown under four foliar application levels of N and Zn at the tillering stage and *OsGS1;1* in the nodes under foliar application at the panicle initiation stage. (**B**) The relative gene expression level of *OsFd-GOGAT* in the stems under foliar application at the tillering stage and *OsFd-GOGAT* in panicles under foliar application at the panicle initiation stage. Each result represents the average of three independent replications. Different letters above the bars indicate significant difference effect of foliar applications of N and Zn by LSD 0.05 at *p* < 0.01. Bars are standard errors of means (SE) in each treatment (n = 3).

The expression of the glutamine synthetase gene (*OsGS1;1*), a marker for N metabolism, was significantly elevated by 84.15-fold in leaves by N+Zn+ treatment at the tillering stage (Figure 4A). Conversely, *OsGS1;1* expression was higher in the nodes under N+Zn0 at the panicle initiation stage. The gene *OsFd-GOGAT*, involved in nitrogen metabolism and chlorophyll synthesis, displayed varying expression patterns, being decreased in leaves with N+Zn0, N0Zn+, and N+Zn+ at the tillering stage but increased in the stems with N0Zn+ and N+Zn+ application; at the panicle initiation stage, expression levels were increased in leaves, stems, and panicles by both N+Zn0 and N+Zn+ treatments (Figure 4B). 

Collectively, our findings reveal that the combined N+Zn+ application enhances the uptake and utilization of these nutrients, with significant upregulation of genes related to Zn and N metabolism, thus providing insights into the complex synergistic interactions between nutrient application and gene expression in rice plant physiology upon foliar nutrient supply.

## 3. Discussion

### 3.1. Foliar Application of Nitrogen and Zinc Fertilizers Improves Yield and Nutrient Accumulation in Rice

Foliar fertilization is increasingly favored due to its efficiency, reduced fertilizer rate, less contamination, and targeted application [13] N and Zn are well known as critical fertilizers for rice crop production; N enhances the uptake and remobilization of Zn, with foliar N+Zn+ improving rice yields and nutrient uptake [14]. Studies have shown the effectiveness of foliar N and Zn on rice yields. For example, the Zn application can boost tiller number, tiller length, seed count, and 1000-seed weight [15], whereas Khampuang et al. [16] observed no significant improvement in yield from foliar N. These discrepancies may be due to variations in crop species, varieties, soil fertility, and application techniques.

Our study found that foliar application of N+Zn+ at the tillering stage significantly enhanced grain yield, aligning with previous results. This boost in yield was linked to the improvements in yield components: tiller and panicle numbers per plant, spikelets per panicle, and filled spikelets. These three components are produced from during the vegetative to the early reproductive phase and thus could explain the phenomenon of higher yield when treated with N+Zn+ at the tillering stage. During the tillering stage, the net photosynthetic and transpiration rates of rice plants are higher compared to the panicle stage [17], and the proper N and Zn application at this stage helps to maximize photosynthetic capacity and yield potential by increasing the leaf area index (LAI) [18]. Zhao et al. [19] also noted that ZnSO_4_ application improved plant growth by enhancing chlorophyll levels and photosynthesis, leading to a higher LAI. Thus, timely foliar application of N+Zn+ at tillering is crucial for maximizing key attributes related to grain yield, e.g., tiller and panicle numbers and spikelet number per panicle.

Foliar N and Zn application influenced the grain Zn and N concentrations. Foliar N and Zn applied at the tillering stage did not enhance grain Zn levels, likely due to the early timing of application, when the seeds had not yet developed, and thus may not have aligned with grain nutrient translocation and accumulation. Other studies have also suggested that combined N and Zn application can improve grain yield but decrease grain Zn concentrations due to the dilution effects of higher productivity [20,21]. Therefore, the amount of Zn absorbed by the grains per plant was calculated by multiplying grain yield by grain Zn concentration for a more accurate evaluation of the effect of foliar N and Zn fertilization on grain Zn accumulation (also known as total grain Zn yield). Foliar N0Zn+ and N+Zn+ increased the grain Zn content when applied both at tillering and panicle initiation stages. These results confirmed those of a previous investigation showing that for the evaluation of the grain nutritional effects of fertilizer treatments, changes in grain concentrations of nutrients should also be taken into consideration together with grain yield. Conversely, applying N and Zn at the panicle initiation stage improved the grain Zn concentration compared to the tillering stage, consistent with the findings of Hussain et al. [22]. Thus, applying foliar Zn either individually or in combination with N at the panicle initiation stage allowed Zn absorption through the leaves and remobilized into the grains, the primary sink of rice plants, during the seed setting and filling periods. However, a previous study reported that the solubility of Zn forms impacts the absorption capacity, with nano-ZnO particles potentially offering longer-term solubility compared to the conventional ZnSO_4_ [23,24]. In addition, ZnSO_4_ may also be less effective due to the precipitation issues, leading to higher Zn absorption and concentrations (26–29 mg Zn/kg) when using soluble Zn forms. This study confirmed that applying N+Zn+ at the panicle initiation stage improved grain Zn concentration compared to the tillering stage, indicating that foliar Zn application at panicle initiation is more effective for remobilizing and accumulating Zn into grains.

Grain Zn loading is primarily due to Zn remobilization from the source tissues [25]. Increased grain yield with foliar N0Zn+ and N+Zn+ indicates effective Zn utilization. Additionally, foliar N application, with or without Zn, can enhance the grain N concentration, further boosting grain Zn levels in indica rice varieties. The positive correlation between Zn and N concentrations in this study supports previous findings [26,27]. The Zn-N correlation holds under adequate Zn availability, although non-foliar Zn fertilizers in low Zn soils disrupt this correlation [14]. Foliar applied fertilizer was suggested as a potential method to improve both yield and grain nutrient concentration, especially the latter compared to soil fertilizer application, as the impact of the fertilizer use strategy on grain micronutrient concentration was much more pronounced, for example, Zn in rice plants grown under field conditions [28,29]. The higher agronomic effectiveness of foliar fertilizer application over soil application in boosting grain Zn concentration has also been shown for several other crops [1,28].

### 3.2. Foliar Application of Nitrogen and Zinc Fertilizers Effects on the Related Genes Expression

Foliar-applied Zn is absorbed by the leaf epidermis and transported to the grains via phloem; the process is regulated by Zn transporters [29]. In our study, foliar N+Zn+ at tillering upregulated the expression of *OsZIP3*, *OsZIP4*, and *OsZIP9*, genes that are crucial for Zn distribution and utilization. The treatment enhanced cell metabolism and stress tolerance, leading to increased yield. *OsZIP3* is a key Zn transporter, while *OsHMA2* is involved in unloading Zn from the xylem [30]. *OsZIP4* helps regulate the Zn supply and transport to young leaves [14] and is vital for photosynthesis and Zn transport to tiller buds [31].

The results concerning gene expression varied at the panicle initiation stage. Higher expression of *OsZIP4*, *OsZIP7*, *OsZIP9*, *OsHAM2*, *OsDUR3*, *OsAAP1*, *OsGS1;1*, and *OsFd-GOGAT* was observed in response to foliar N+Zn0, indicating specific roles in nodes, where nutrient and ion transport is crucial [32]. *OsAAP1* and *OsZIP7* are involved in amino acid and Zn transport, respectively [33,34]. Foliar N+Zn+ application led to increased expression of genes involved in Zn translocation from stems to leaves and panicles, enhancing grain Zn and N concentrations.

This study has demonstrated the synergistic effect of N and Zn foliar application on yield and grain Zn accumulation in rice. The foliar application of N+Zn+ at the tillering stage significantly increased grain yield compared to the control. Additionally, grain Zn concentrations increased significantly when N0Zn+ and N+Zn+ were applied at panicle initiation. The gene expression analysis confirmed that applying N and Zn at the appropriate times significantly influenced rice productivity and grain Zn concentration. At the tillering stage, applying foliar N+Zn+ significantly elevated the expression of Zn transporters such as *OsZIP3*, *OsZIP4*, and *OsZIP9* in leaves, thereby enhancing grain yield. This indicates that the timing of nutrient application significantly affects the expression of genes involved in zinc and nitrogen transport and accumulation in rice. The elevated expression of the zinc transporters *OsZIP3*, *OsZIP4*, and *OsZIP9* at the tillering stage suggests that this period is crucial for enhancing zinc uptake, a process that is vital for the plant’s growth and development. Interestingly, applying foliar nutrients at the panicle initiation influenced a broader range of genes, including *OsZIP4*, *OsZIP9*, *OsHAM2*, *OsDUR3*, *OsAAP1*, *OsGS1;1*, and *OsFd-GOGAT*, affecting grain Zn and N accumulation. The broader gene expression observed at the panicle initiation stage highlights a more complex response, indicating that both N and Zn are critical for preparing the plant for grain development. This stage involves multiple genes that facilitate not only Zn uptake (through transporters) but also N assimilation and metabolism. The results suggest that effective nutrient management must be tailored to specific growth stages to maximize both nutrient efficiency and crop yield.

To further enhance our understanding and management of nutrient application, we should consider moving from a stage-specific approach to a cell-specific strategy. With advancements in technologies such as single-cell transcriptomics and spatial genomics, we can determine how individual cell types within rice plants respond to N and Zn at various developmental stages. This shift would enable us to tailor nutrient applications more precisely, targeting specific cells responsible for nutrient uptake and metabolism. By focusing on the cellular mechanisms driving nutrient efficiency, we can optimize crop yield and quality even further, paving the way for more sustainable agricultural practices. Additionally, future studies should explore the potential of advanced foliar application methods such as drones or automated sprayers and assess cost-effective formulations, including slow-release and nano-fertilizers. These advancements would enable farmers to adopt the most feasible practices, maximizing both efficiency and benefit.

## 4. Materials and Methods

### 4.1. Experimental Design and Rice Cultivation

A factorial experiment was arranged in a completely randomized design with three independent replications, each consisting of two pots, resulting in a total of 48 pots. One pot in each replicate was employed for gene expression analysis, and the other was used to assess yield and the concentrations of Zn and N. The experiments were carried out in a controlled environment chamber with a 14/10 h light/dark cycle, 200 µmol photons m^2^/s, a temperature of 28/25 °C, and 80% relative humidity. Plastic pots (4 inches in diameter and 5 inches deep) were filled with 0.25 kg of soil containing nitrate-N 28.0 ppm, ammonium-N 3.9 ppm, phosphorus 4.0 ppm, potassium 66.0 ppm, calcium 210.0 ppm, magnesium 80.0 ppm, sodium 56.0 ppm, and chloride 45.0 ppm, with a soil pH of 5.9. Seven-day-old indica rice seedlings were transplanted into the pots, one seedling per pot, and maintained under waterlogged conditions. The foliar application of N and Zn fertilizers is used with urea and zinc sulfate. The treatments were as follows: (1) no N and Zn (N0Zn0), (2) foliar N with no Zn (N+Zn0), (3) no foliar N with foliar Zn (N0Zn+), and (4) foliar N and Zn (N+Zn+). Foliar N was applied as 1% urea and Zn as 0.5% ZnSO_4_ at a rate of 1000 L/ha. The control (N0Zn0) was sprayed with distilled water. A 1.25 L compression sprayer was used to ensure uniform application of the solutions to the leaves, minimizing cross-contamination between pots. Fertilizers were applied twice: at the tillering stage and panicle initiation. Foliar treatments at the tillering stage were based on plant growth, and the panicle initiation stage occurred after the maximum tiller number.

### 4.2. Analytical Procedures

At maturity, the samples were harvested, and seeds were separated from straw manually. Evaluation criteria included tiller per plant, panicle per plant, spikelet number per panicle, and percent filled grains. Paddy rice was cleaned, air-dried, and weighed to determine grain yield. The straw was air-dried to measure dry weight. The husks were removed from the grains to produce brown rice. Leaf and brown rice samples were oven-dried at 75 °C for 72 h, then ground to powder for N and Zn concentration analysis.

Zn concentration in plant tissues was expressed as mg/kg dry matter and measured by atomic absorption spectrophotometry (AA) model control 800 (Analytika Jena AG, Jena, Germany). Approximately 0.5 g of ground sample was dry-ash burned in a muffle furnace at 535 °C for eight hours. The ash was acid-extracted with HCl (1:1) for 20 min. Certified reference materials (SRM 1547) from the National Institute of Standards and Technology were used for accuracy checks. Grain Zn content was calculated by multiplying grain Zn concentration by grain yield, expressed in mg per plant dry weight.

Total N concentration was determined by combustion analysis using a LECO combustion furnace (LECO Co., St Joseph, MI, USA). Samples were weighed into a boat or foil cup and combusted in a high-temperature chamber with oxygen flow, converting elemental carbon, nitrogen, and sulfur into CO_2_, SO_2_, N_2_, and NO_x_. The gases were analyzed using infrared absorption and thermal conductivity detection to calculate the percentage of total N.

### 4.3. Quantitative Real-Time Polymerase Chain Reaction (RT-qPCR)

Seven days post foliar application, the youngest emerged leaf blade (YEB) of the main stem, and the upper node (3 cm in length) were sampled for gene expression analysis at both tillering and panicle initiation stages. Panicles (3 cm in length) were also sampled at panicle initiation. RT-qPCR was performed to assess gene expression in response to foliar N and Zn application. Specific primers were designed for the genes *OsZIP3*, *OsZIP4*, *OsZIP5*, *OsZIP7*, *OsZIP9*, *OsHMA2*, *OsDUR3*, *OsAAP1*, *OsGS1;1*, and *OsFd-GOGAT* (Table 1). Total RNA was extracted using the E.Z.N.A.^®^ Plant RNA Kit (Omega BIO-TEK, Norcross, GA, USA), treated with TURBO DNA-free™ Kit (Carlsbad, CA, USA), and reverse transcribed into cDNA using iScript cDNA Synthesis Kits (BioRad, Hercules, CA, USA). RT-qPCR was conducted in 384-well plates with at least three replicates per sample using the QuantStudio 7 Q-PCR system (Genomics Core, MSU, East Lanising, MI, USA). Reactions were performed in a 10 µL volume with 5 µL of SsoFast EvaGreen Supermix (BioRad, CA, USA), 1 µL of template cDNA, 3 µL of distilled water, and 1 µL of each forward and reverse primers [35,36].

### 4.4. Statistical Analysis

The data were analyzed using analysis of variance (ANOVA) with Statistix 8 (Analytical Software, SXW). A least significant difference (LSD) test at *p* < 0.05 was used to compare treatment means. Linear regression was employed to describe the relationships between grain yield, straw yield, and Zn and N concentrations in the grains.

## 5. Conclusions

In summary, combining urea (N) with ZnSO_4_ (Zn) in biofortification programs effectively improves rice yield and grain Zn and N concentrations. Foliar application of N+Zn+ at the tillering stage enhances yield and gene expression, while application at the panicle initiation stage boosts grain Zn and N concentrations through improved gene expression related to Zn transport and utilization. The results from this study confirm the synergistic interaction between N and Zn through N and Zn transporter-relate genes. These results will be very useful for farmers and consumers wishing to improve productivity and nutritional intake, respectively, especially given the emerging need to understand the nutrient homeostasis interaction under climate change scenarios [46,47].

## Figures and Tables

**Table 1:** Gene-specific primers used in real-time QPCR analysis.

Gene Name	Primer Sequence (5′ → 3′)	Reference
*OsZIP3*	forward AAAAAGCAGGCTTCTCATCATCTTATTCCCTTCTAC	[37]
	reverse AGAAAGCTGGGTCTGTGGTGTTAGCACAGTCGC	
*OsZIP4*	forward CACCATGGACGCCATGAGGCAGAGCACGCG	[38]
	reverse TCATGCCCATATGGCAAGCAGAGACATCAT	
*OsZIP5*	forward CATGAAGACCAAGGTGCAGAGAAGG	[37]
	reverse TCACGCCCAGATGGCGATCA	
*OsZIP7*	forward TGTCCGATGGAGCGGTTCG	[39]
	reverse CCTCTACATTAGTCCCTGAG	
*OsZIP9*	forward ATCTTCTTCTCGCTAACCACAC	[37]
	reverse GCAGCCGCTGCGTCGAGAAT	
*OsHMA2*	forward CATAGTGAAGCTGCCTGAGATC	[40]
	reverse GATCAAACGCATAGCAGCATCG	
*OsDUR3*	forward CCTTGGCTACTTCACGCTGT	[41]
	reverse TGCATCTCCGTCTCGTGTAG	
*OsAAP1*	forward CCCATTACCACCTCCACCTC	[42]
	reverse ACCTTCTCTTGCGGCCTCTC	
*OsGS1;1*	forward ACCTCCTCCAGAAGGACAT	[43]
	reverse GTGCCTGAGCTTGAGCTTCT	
*OsFd-GOGAT*	forward GCATACTTGTGAAGCACCGAAGTG	[44]
	reverse CTGCAAATAGCAACCTAGCGTCAG	
*α-tubulin*	forward TCTTCCACCCTGAGCAGCTC	[45]
	reverse AACCTTGGAGACCAGTGCAG	

## Data Availability

The datasets and plant materials generated and analyzed during this study are available from the corresponding authors H.R. and C.P.-U.-T. upon request.
